# Distinction of phyllodes tumor from fibroadenoma: Cytologists’ perspective

**DOI:** 10.4103/0970-9371.70739

**Published:** 2010-04

**Authors:** Ranjana Bandyopadhyay, Dipanwita Nag, Santosh Kumar Mondal, Subhalakshmi Mukhopadhyay, Sumit Roy, Swapan Kumar Sinha

**Affiliations:** Department of Pathology, Medical College, Kolkata, India

**Keywords:** Cytopathology, fibroadenoma, phyllodes tumour, stromal fragment

## Abstract

**Background:**

Fibroadenomas and phyllodes tumors may have similar cytological appearances. However, a detailed study of cytomorphology of stromal elements may be helpful in differentiation.

**Aim:**

To evaluate the cytological features of phyllodes tumor in our study with special reference to features that can help distinguishing it from fibroadenoma.

**Materials and Methods:**

The archival materials of our hospital were searched from January 2006 to January 2009 for histopathologically-diagnosed cases of phyllodes tumor. The cases in which previous cytopathology smears were available were included in the study. The cytomorphology of 10 such cases were compared with 25 cytologically-diagnosed and histopathologically-confirmed cases of fibroadenoma.

**Results:**

The size, cellularity of stromal fragments, and the proportion of spindle cells in the background are important features in such differentiation.

## Introduction

Phyllodes tumor is a rare fibroepithelial neoplasm accounting for less than 1% of all breast tumors.[1] This tumor is said to be underdiagnosed by the pathologists and undertreated by the surgeons.[2] Thus, the preoperative diagnosis of this tumor becomes very important to allow correct surgical planning and avoid reoperation (wide local excision with at least 1 cm margin is currently the treatment of choice for phyllodes tumor). Fibroadenomas and phyllodes tumor may have identical clinical and radiological appearance. Though age at presentation may give some diagnostic clue, it should not be overemphasized. Preoperative fine needle aspiration (FNAC) of the lesion becomes important to offer tissue diagnosis. To compound the diagnostic difficulties, fibroadenomas and phyllodes tumor have many cytological features in common. The cytological features of phyllodes tumor have been documented in the literature.[3–5] But the studies that have tried to compare the cytological features of these two tumors have given conflicting results. Although the diagnosis of malignant phyllodes tumor is not difficult, the diagnosis of low grade phyllodes tumor and its distinction from fibroadenoma on FNAC becomes difficult due to overlapping features between the two lesions.[6] However, some investigators have suggested features that point in favor of phyllodes tumor.[5,7,8] With this background, we tried to evaluate the cytological features of phyllodes tumor in our study with special reference to features that can help in distinguishing it from fibroadenoma.

## Materials and Methods

The archival files of Medical College Hospital were searched from 2006 to 2009 for histopathologically diagnosed cases of phyllodes tumor and 18 such cases were found, of which preoperative FNAC smear were available in 10 cases. The histopathology slides of these 10 cases were retrieved and reviewed to confirm the diagnosis. Only histopathologically confirmed cases of phyllodes tumor were included in the study. There were eight benign, one borderline and one malignant phyllodes tumor. The control group comprised 25 cytologically-diagnosed and histologically-confirmed cases of fibroadenoma. The cytological materials in all cases comprised air-dried May-Grünwald-Giemsa (MGG) stained and alcohol-fixed Papanicolaou-stained smears. The cytologic features of their epithelium and stromal components and the characteristics of the individual stromal cells constituting the background dispersed cell population were evaluated in detail. The epithelial cell clusters were studied for the following features: number of clusters and sheets, and architecture (normal, hyperplastic, or showing metaplasia). Stromal fragments were assessed for number of fragments (more than 5 or less than 5), cellularity (graded as 1+, 2+, 3+), presence of atypia in stromal cells, and blood vessels crossing stromal fragments. The background stromal cells were also studied in detail and the cellularity was graded as 1+, 2+, or 3+. The characteristics of the dispersed cells were studied and the percentage of the spindle cells noted. Presence of atypia and the giant cells within the dispersed cell population was also noted.

## Results

Cytological features assessed for each tumor type are presented in Table 1. The features in both the tumor groups were characterized by dimorphic pattern. However the only case of malignant phyllodes did not have epithelial component in the FNAV smear and a differential diagnosis of stromal sarcoma was given. Six of the 25 fibroadenomas and 1 out of 10 phyllodes tumor did not show stromal fragment in FNA material.

**Table 1:** Comparison of cytological characteristics between phyllodes tumour and fibroadenomas

Characteristic	Phyllodes tumor (%)	Fibroadenoma (%)
Epithelial fragments		
Number		
Few (<5)	2 (20)	2 (8)
Many (>5)	8 (80)	23 (92)
Characteristics		
Hyperplasia	2 (20)	5 (20)
Nuclear atypia	0	0
Apocrine metaplasia	1 (10)	2 (8)
Stromal fragments		
Number		
Absent	1 (10)	6 (24)
Few (<5)	2 (20)	8 (32)
Many (>5)	7 (70)	11 (44)
Cellularity		
1 +	1 (10)	20 (80)
2 +	3 (30)	3 (12)
3 +	6 (60)	2 (8)
Blood vessels crossing fragments	3 (30)	5 (20)
Dispersed cell population		
Cellularity		
1+	3 (30)	8 (32)
2+	4 (40)	15 (60)
3+	3 (30)	2 (8)
Giant cells	1 (10)	0
Spindle cells		
<10%	1 (10)	21 (84)
10–30%	4 (40)	4 (16)
>30%	5 (50)	0

**Figure 1:**
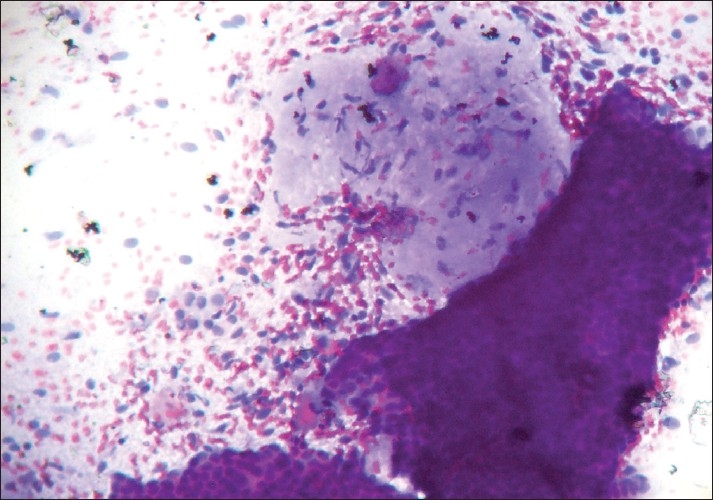
Photomicrograph showing monolayered sheets of epithelial cells with myoepithelial cells within the clusters and fair number of bare nuclei in the background, and a stromal fragment with low cellularity and ill-defined margin (PAP, ×200)

Epithelial features did not vary much between the two tumor groups apart from more abundance of epithelial cells in fibroadenomas. Epithelial hyperplasia was noted in both the groups. The fibroadenomas commonly showed typical branching monolayered sheets of epithelial cells with myoepithelial cells within the clusters, and variable numbers of bare nuclei in the background [Figure 1].

The number of stromal fragments varied but was in the lower range compared to phyllodes tumors. The stromal fragments were typically hypocellular with myxoid appearance and poorly-defined margins. However, two of the 25 cases of fibroadenomas showed stromal hypercellularity (3+) and all of these cases were under 20 years of age. On the other hand, the cellularity of stromal fragments as well as the presence of atypia in the stromal cells was found more frequently in the phyllodes tumors. Compared to fibroadenomas, the stromal fragments of phyllodes tumors were larger and hypercellular. The stromal fragments of phyllodes tumor showed well defined borders (6/10) compared to fibroadenomas (7/25).

**Figure 2:**
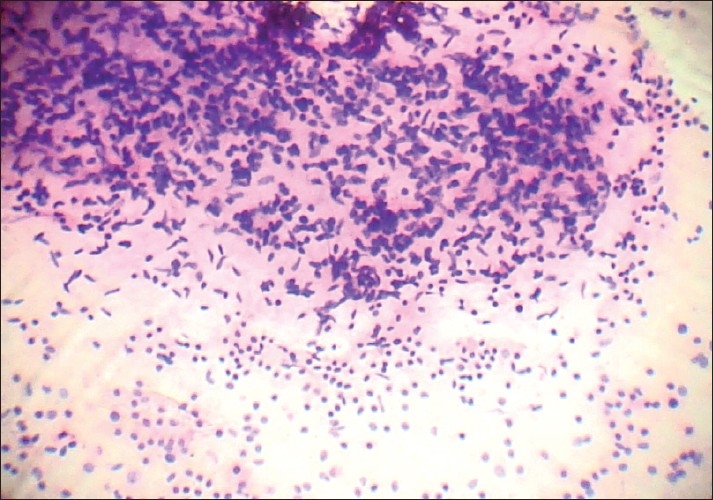
Photomicrograph showing hypercellular stromal fragment – graded as 3+ cellularity (PAP, ×200)

Strands of myxoid material were noted in seven cases of phyllodes tumor (70%). Sixty percent of phyllodes tumor showed 3+ stromal cellularity [Figure 2] and 50% showed >30% spindle cells in the dispersed population.

**Figure 3:**
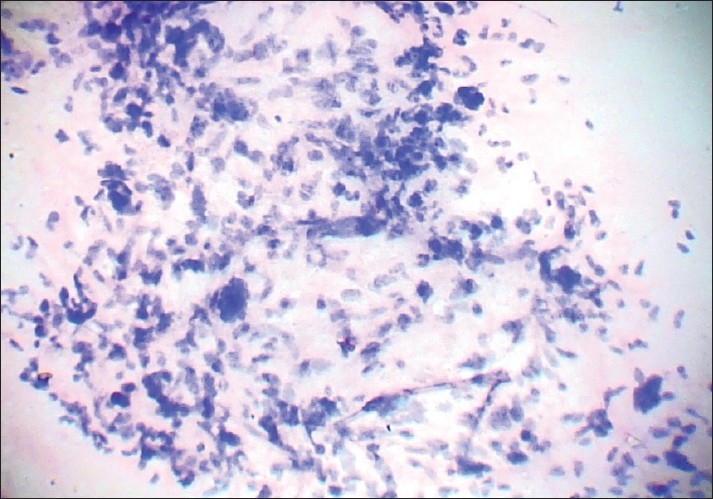
Photomicrograph showing stromal cellular atypia with some bizarre cells in a case of malignant phyllodes tumour. A benign epithelial fragment is visualized in the lower part (PAP, ×400)

In case of malignant phyllodes, the cytological features of malignancy were distinctly evident [Figure 3]. We encountered two cases of recurrent phyllodes tumor and cytological features were similar to those at presentation in one case, while the other showed increased cellularity and atypia in the recurrence.

In the background cellular elements, both fibroadenomas and phyllodes tumors showed predominance of round to oval nuclei. There was marked overlap in the degree of background cellularity in the two groups. However, the number of spindle cells in the background was more in cases of phyllodes tumors

**Figure 4:**
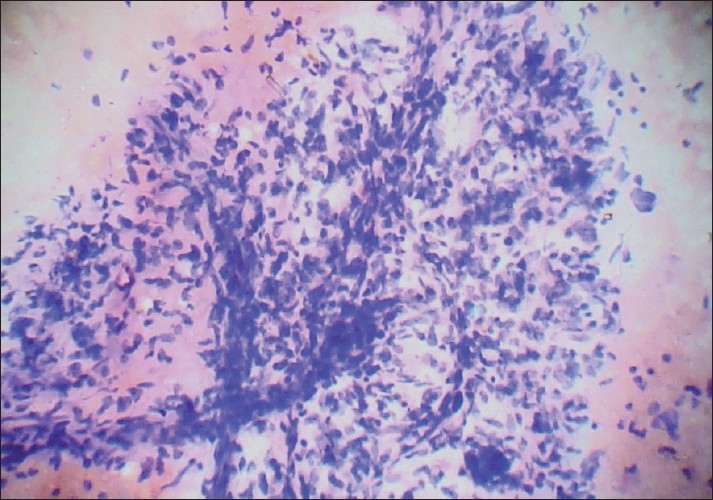
Photomicrograph showing blood vessels crossing the hypercellular stromal fragment (PAP, ×400)

Blood vessels crossing stromal fragments were found rarely in our study [Figure 4] and were present in both the tumor groups. Hence, it was not found useful in differentiation.

## Discussion

Cytological features of phyllodes tumors have been well characterized. Criteria for the diagnosis of benign phyllodes tumor include at least two large stromal fragments, hypercellular fragments and moderate to large number of dissociated stromal cells.[9] Fibroadenomas and phyllodes tumors share a dimorphic pattern with both epithelial and stromal components. The distinguishing features relate to the stroma, including the presence of hypercellular stromal fragments,[3–5,8,10,11] cellularity of background nuclei,[12] and cellular composition and morphology of background nuclei.[4,11,13] Although there have been a number of studies documenting the cytological features of phyllodes tumor, few studies have compared the cytomorphology of phyllodes tumor with fibroadenoma and these have yielded conflicting results.[3,5,8,10,11,14]

Scolyer *et al*.[15] proposed that the presence of hypercellular stromal fragments is the most useful in distinguishing phyllodes tumor from fibroadenomas and the presence of nuclear atypia is useful in separating malignant phyllodes tumor from benign ones. Hypercellularity of stromal fragments have been noted in both benign phyllodes tumors and fibroadenomas and some authors have suggested that stromal cellularity should not be considered as the cytological differentiating feature.[11] However most workers have noted hypercellular stromal fragments are more frequent in phyllodes tumor compared to fibroadenoma.

In our study, we found stromal fragments of phyllodes tumor to be larger and hypercellular with more well-defined borders compared to fragments in fibroadenomas. Hypercellular stromal fragments may also be present in some cases of fibroadenoma. In these cases, the degree of background cellularity, the composition of these cells and the epithelium to stroma ratio may be useful features in differentiation. Studies comparing the cellularity of dispersed stromal cells between phyllodes tumor and fibroadenoma have shown that phyllodes tumor have more 1+ and fewer 2+ cellularity than fibroadenoma and 3+ cellularity was similar in both the groups. In our study, however, we found 3+ cellularity to be more common in phyllodes tumors than fibroadenoma (30% compared to 8%). The importance of spindle cells in the dispersed cell population has been emphasized by some workers,[4,6,13] and we found it to be useful in practice. This feature seemed particularly helpful in differentiating from cellular fibroadenoma. While testing these parameters individually may not give encouraging results, taken together, they can be used effectively in distinguishing these two groups of tumors.

Different studies have suggested epithelial features such as folded sheets, blunt sheets may be important in differentiation but in our study no epithelial feature was found useful in this regard. The important cytological feature of borderline and malignant histpathological subgroup of phyllodes tumor is the presence of abundant hypercellular stromal fragments that show variation in nuclear size and shape.[4,13] There was only one case of malignant phyllodes in our series which showed obvious features of stromal hypercellularity, atypia and bizarre cells. There was significant number of tumor giant cells in the background. The case of borderline phyllodes tumor did not show any cytological clue to its nature and appeared similar to a benign tumor. Studying the histology we could appreciate that the cellular atypia was only focal in this case and hence was not properly represented in the FNAC materials. Hence it is important to know that stromal overgrowth and other features of malignancy may be present focally requiring FNAC from multiple sites to document malignancy.[15] The diagnostic difficulty is often compounded by sampling problems which is important because of heterogeneous nature these tumors.

The improvements in core biopsy guns and refinements of image-guided techniques for sampling breast lesions have resulted in rapid acceptance of core needle biopsy in the preoperative diagnosis of breast lumps. Whether it should be used in conjunction with FNAC or should replace, it is a matter of debate. But core biopsy in cases of phyllodes tumor definitely helps in better diagnostic accuracy as the hurdle of sampling problem is overcome to some extent by acquiring multiple cores.
